# Lack of Association Between DJ-1 Gene Promoter Polymorphism and the Risk of Parkinson’s Disease

**DOI:** 10.3389/fnagi.2019.00024

**Published:** 2019-02-26

**Authors:** Lu He, Suzhen Lin, Hong Pan, Ruinan Shen, Mengyan Wang, Zhihao Liu, Shiyao Sun, Yuyan Tan, Ying Wang, Shengdi Chen, Jianqing Ding

**Affiliations:** ^1^Department of Neurology, Institute of Neurology, Ruijin Hospital, Shanghai Jiao Tong University School of Medicine, Shanghai, China; ^2^Laboratory of Neurodegenerative Diseases, Institute of Health Science, Shanghai Institutes for Biological Sciences, Chinese Academy of Sciences, Shanghai Jiao Tong University School of Medicine, Shanghai, China; ^3^Shanghai Jiao Tong University School of Medicine, Shanghai, China; ^4^The University of Melbourne, Melbourne, VIC, Australia

**Keywords:** PARK7/DJ-1, promoter, polymorphism, Parkinson’s disease, eQTL

## Abstract

Low DJ-1 protein level caused by DJ-1 gene mutation leads to autosomal recessive Parkinson’s disease (PD) due to impaired antioxidative activity. In sporadic PD patients, although mutations were rarely found, lower DJ-1 protein level was also reported. Dysregulation of DJ-1 gene expression might contribute to low DJ-1 protein level. Since the promoter is the most important element to initiate gene expression, whether polymorphisms in the DJ-1 promoter result in the dysregulation of gene expression, thus leading to low protein level and causing PD, is worth exploring. The DJ-1 promoter region was sequenced in a Chinese cohort to evaluate possible links between DJ-1 promoter polymorphisms, PD risk and clinical phenotypes. Dual-luciferase reporter assay was conducted to evaluate the influence of promoter polymorphisms on DJ-1 transcriptional activity. Related information in an existing genome-wide association studies (GWAS) database were looked up, meta-analysis of the present study and other previous reports was conducted, and expression quantitative trait loci (eQTL) analysis was performed to further explore the association. Three single nucleotide polymorphisms (SNPs) (rs17523802, rs226249, and rs35675666) and one 18 bp deletion (rs200968609) were observed in our cohort. However, there was no significant association between the four detected genetic variations and the risk of PD either in allelic or genotype model, in single-point analysis or haplotype analysis. This was supported by the meta-analysis of this study and previous reports as well as that of GWAS database PDGene. Dual luciferase reporter assay suggested these promoter polymorphisms had no influence on DJ-1 transcriptive activity, which is consistent with the eQTL analysis results using the data from GTEx database. Thus, DJ-1 promoter polymorphisms may play little role in the dysregulation of DJ-1 expression and PD susceptibility in sporadic PD.

## Introduction

Parkinson’s disease (PD) is a common neurodegenerative disorder affecting approximately 1% of people over the age of 60 of the world and 1.7% of people over 65 in China (Zhang et al., 2005; de Lau and Breteler, 2006). Clinically, PD is manifested by classical motor symptoms, including tremor, rigidity, bradykinesia, and postural instability (Kalia and Lang, 2015). Increasing evidence has suggested that PD is probably caused by a combination of genetic and environmental risk factors (Kalia and Lang, 2015). During the past 20 years, more than 20 locis and 9 genes have been found associated with PD (Kalinderi et al., 2016). One such gene, PARK 7/DJ-1 aroused our interest since it played an important role in both familial and sporadic PD.

The human DJ-l gene is located on 1p36.23. DJ-1 protein acts as a molecular chaperone which plays a protective role in oxidative stress (Canet-Aviles et al., 2004). Loss of function mutations in the DJ-1 gene, including deletion of exon 1-5 (Bonifati et al., 2003), L166P (Bonifati et al., 2003), R98Q (Abou-Sleiman et al., 2003; Hague et al., 2003), M26I (Abou-Sleiman et al., 2003), E64D (Hering et al., 2004), and L172Q (Taipa et al., 2016), have been demonstrated to cause degeneration of dopamine neurons and autosomal recessive inherited PD. However, these causative mutations explained less than 10% of PD patients since about 90% of cases are sporadic without these mutations (Sutherland et al., 2009). Shen’s group examined DJ-1 protein levels in SNc of 18 sporadic PD cases and found a lower level than that of normal control group (Nural et al., 2009). Similarly, lower DJ-1 protein level was also observed in cerebrospinal fluid (CSF) of sporadic PD patients compared with normal controls (Hong et al., 2010). These findings strongly indicated that a low level of DJ-1 might contribute to the pathogenesis of sporadic PD. The observation in our previous research that knockdown (KD) DJ-1 could increase MPP+ induced ROS production and cell death further supported the above hypothesis (Wang et al., 2011). However, the mechanism of low DJ-1 protein level in sporadic PD is still obscure.

**FIGURE 1:**
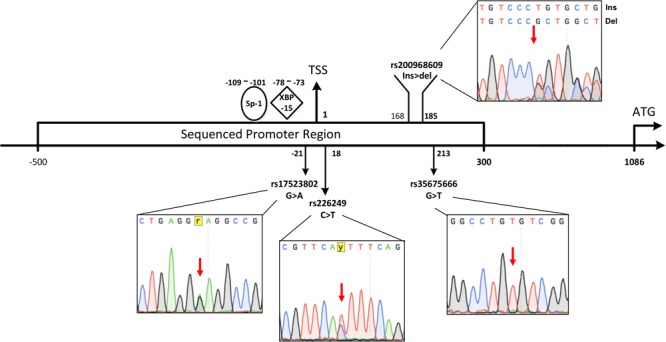
Schematic view of relative positions of the polymorphisms investigated in the study. The first base of TSS was defined as 1, the first upstream base of TSS is –1, the relative positions of 4 polymorphisms observed in our cohort and previous reported functional sites (Sp-1 or XBP-1S binding site) were calculated based on TSS. The range of –500∼+300 across TSS (NC_000001.11: 7961201-7962000) containing the above functional areas of DJ-1 promoter was sequenced in the study. Representative sequence of rs17523802 G > A heterozygote, rs226249 heterozygote, rs200968609 heterozygote, and rs35675666 homozygote were shown.

Since DJ-1 gene mutations are rarely found in sporadic PD, we hypothesized that low DJ-1 protein levels in sporadic PD might be related to dysregulation of gene expression. The transcriptional initiation is the basic step of gene expression, and the promoter is the most fundamental element to initiate transcription. Polymorphic sites in the promoter may affect the binding and regulatory ability of transcription factors (TFs) to the promoter and influence transcriptional activity. Whether polymorphisms in the DJ-1 promoter affect the transcriptional activity and relate to the occurrence of PD is unknown. The promoter of DJ-1 is believed in a 2.1 kB area (-1015∼+1104) across the Transcription Start Site (TSS) (Taira et al., 2001). Taira et al. (2001) discovered a significant regulatory region in the promoter present at -109 to -101 from the TSS, and Duplan et al. (2013) showed a region located at -78 to -73 from the TSS (Figure 1), which could dramatically upregulate the expression of DJ-1. Considering that polymorphisms present at or near these regions might affect promoter activity and lead to low DJ-1 protein levels, we sequenced an area containing the above regions of DJ-1 promoter (NC_000001.11: 7961201-7962000) in 523 sporadic PD patients and 599 controls in Chinese Han population to screen the polymorphisms which may be associated with PD. To further analyze the genetic association, detected polymorphisms in the DJ-1 gene promoter region were looked up in existing public genome-wide association studies (GWAS) meta-analysis database PDGene. Meta-analysis of this study and previous reports was also conducted. Dual-luciferase assay was used to access the influences of detected polymorphisms on DJ-1 transcriptional activity. To further assess the association between detected polymorphisms and human brain DJ-1 expression level, expression quantitative trait loci (eQTL) analysis results were searched in GTEx Portal.

## Materials and Methods

### Study Population

523 PD patients were recruited from movement disorders clinics in Ruijin Hospital, Shanghai, China. All patients were diagnosed with idiopathic PD by at least two movement disorders specialists according to the United Kingdom Parkinson’s Disease Society (UKPDS) Brain Bank Clinical Diagnostic Criteria (Gibb and Lees, 1988). 599 unrelated controls were recruited from communities of Shanghai in epidemiologic investigation program. Each of the control had no evidence of neurodegenerative disease. All participants were Chinese Han residing in Shanghai. Any participants with a family history of PD were excluded. Demographic information [gender, age, age at onset, medication status, oral Levodopa Equivalent Dose (LED), disease duration, etc] and peripheral blood samples were collected from participants. The modified Hoehn and Yahr scale (H-Y) was rated in the OFF state of each patient. LED was computed according to the protocol provided by Tomlinson et al. (2010). Lifestyle factors including smoking and consumption of alcohol were also recorded. PD patients were divided into Tremor Dominant (TD), Akinetic/Rigid (AR) and Mixed (MX) subtypes by the criteria used in previous studies (Rajput et al., 2008). TD: rest tremor as sustained dominant symptom over bradykinesia and rigidity; Akinetic/Rigid dominant (AR): predominantly bradykinetic motor features with no or only mild rest tremor; Mixed motor feature group (MX): rest tremor, bradykinesia and rigidity present at the time of diagnosis or sustained comparable severity of tremor and bradykinetic motor features. Patients with age at onset <50 were classified as early-onset PD (EOPD), ≥50 years as late-onset PD (LOPD). The study was in accordance with the Helsinki Declaration of 1975. Written informed consents were obtained from all participants. Approval for the study was obtained from the Ethics Committee of Ruijin Hospital, Shanghai Jiao Tong University School of Medicine (2011-No. 13).

### Blood Sample Collection and Genetic Analysis

Peripheral blood samples were collected in EDTA anticoagulant tubes and placed immediately on ice. After the lysis of erythrocytes, blood samples were centrifuged at 3000 rpm for 10 min to isolate leukocytes. DNA was extracted from leukocytes through standardized phenol/chlorine extraction method. The range of -500∼+300 across TSS (NC_000001.11: 7961201–7962000) in DJ-1 promoter was amplified and sequenced (Schematic view of the area was shown in Figure 1). The primers used for polymerase chain reaction (PCR) amplification were as follows: forward 5′-ACTGCTCTAGTCCTGTGGGT-3′ and reverse 5′-CAGCTCGCCTCATGAC-ATCT-3′. With the PrimeSTAR DNA Polymerase (Takara, Dalian, China), following an initial denaturation at 94°C for 5 min, 30 PCR cycles were performed according to a 68–50°C touchdown PCR protocol (the first 12 cycles: 98°C for 15 s, 68–50°C for 15 s, 72°C for 1 min 50 s, the annealing temperature decreases by 1.5°C every cycle from 68 to 50°C; the next 18 cycles: 98°C for 15 s, 50°C for 15 s, 72°C for 1 min 50 s) with a final extension at 72°C for 5 min. After PCR amplification, the product was electrophoresed in 1.5% agarose gels containing ethidium bromide, purified and directly sequenced (Biosune, Shanghai, China). Sequences were aligned to the reference human genome sequence (NC_000001.11) using the SnapGene software (from GSL Biotech, available at ^1^) and the Mutation Surveyor software (form SoftGenetics, available at ^2^).

### Meta-Analysis of the Association Between DJ-1 Promoter Polymorphisms and PD

The four variations were looked up in GWAS meta-analysis database PDGene^3^. Meta-analysis *p*-values and odds ratios (OR) of the four variations based on 13,708 PD cases and 95,282 controls from 13 independent GWAS datasets of European descent were obtained (Nalls et al., 2014). Due to privacy protection and data sharing restrictions, detailed genotype information of the four variations were not accessible. Thus, only Meta *p* values and Meta OR of the four variations in GWAS reports were listed in Table 5. Except for existing GWAS data, other related previous case-control studies were searched in PubMed, Embase and Web of Science, using the following terms “(polymorphism OR SNP) AND (DJ-1 OR PARK7) AND (PD OR Parkinson’s disease),” “(rs17523802 OR rs226249 OR rs200968609 OR rs35675666) AND (PD OR Parkinson’s disease).” Studies on irrelevant polymorphisms were eliminated, and finally, 6 independent case-control studies were selected. Genotype data were retrieved from the 6 studies and analyzed. Detailed information of the studies including race, minor allele frequencies (MAF) and sample counts were shown in Table 6. Meta-analysis was conducted with the Review Manager version 5.3.5 under the random effect model.

### Construction of Luciferase Reporter Gene Vectors and Dual-Luciferase Reporter Assays

The DJ-1 promoter plasmid, containing the T or C allele at rs226249, or the A or G allele at rs17523802 (since rs17523802, rs200968609 and rs35675666 showed strong linkage pattern with *r*^2^ = 1, three haplotypes were constructed: G-C-ins-G and G-T-ins-G to detect rs226249 function, G-C-ins-G and A-C-del-T to detect rs17523802, rs200968609 and rs35675666 function, alleles arrayed in order of rs17523802, rs226249, rs200968609 and rs35675666), were amplified from the genomic DNA of PD patients, using primers containing BglII in the forward primer and HindIII in the reverse primer for cloning (forward: 5′-GAAGATCTACTGCTCTAGTCCTGTGGGT-3′ and reverse: 5′-CCCAAGCTTCATTGCAACCCTGAGATACCCC-3′). PCR was performed: denatured at 94°C for 5 min, and amplified for 30 cycles at 98°C 15 s, 56°C 20 s, 72°C 1 min 45 s, with terminal extension at 72°C 5 min. After digested with Bgl II and HindIII (Takara, Dalian, China) and purified (Tiangen, Beijing, China), the fragments were cloned into the pGL3-basic luciferase plasmid (Promega, Beijing, China).

Human neuroblastoma cells (SH-SY5Y) were cultured in DMEM medium with 10% FBS (GIBCO/Invitrogen, Shanghai, China) and incubated at 37°C in a humidified environment with 5% CO2. SH-SY5Y cells were plated into 24-well culture plates 24 h prior to transfection, and cells were 80% confluent at transfection. 490 ng polymorphism plasmid or pGL3-basic empty plasmid (as a negative control) was transfected into SH-SY5Y cells using Lipofectamine 3000 (Invitrogen, Shanghai, China), with 10 ng Renilla pRL-TK plasmid (Promega, Beijing, China) cotransfected as a normalizing control. After 24 h, cells were rinsed with PBS and harvested with Passive Lysis buffer (Promega). Transcriptional activity was determined using the Dual-Luciferase Reporter Assay System (Promega, Beijing, China) on a Synergy^TM^ H4 Hybrid Microplate Reader (BioTek, Shanghai, China). For each plasmid construct, four independent transfection experiments were carried out and readings were taken in duplicate. The transcriptional activities were reported as relative luciferase activities, which was the ratio of firefly luciferase activities over renilla luciferase activities.

### Analyzing the Effects of DJ-1 Promoter Polymorphisms on DJ-1 Gene Expression in Normal Human Brain

The potential impact of DJ-1 promoter SNPs on DJ-1 gene expression was evaluated by eQTL analysis. The data used for the analyses were obtained from the GTEx Portal^4^ and dbGaP (accession number phs000424.vN.pN).

### Statistical Analysis

All statistical analysis was performed using the SPSS software. For analyzing demographic statistics, a Mann–Whitney *U*-test was used for continuous variables and a Chi-squared test or Fisher’s exact test was used for nominal data. The Chi-squared test or Fisher’s exact test was used to assess the deviation of alleles in (HWE) and to evaluate the differences in genotype and allele distributions between groups. Measures of linkage disequilibrium (D’ and r2) were computed from participants’ genotypes with Haploview 4.1 (from Broad Institute, available at ^5^). Each genotype was estimated by logistic regression analysis presuming additive mode of inheritance under correcting by confounders. A two-tailed *P* < 0.05 was considered statistically significant. For multiple statistical tests, the Bonferroni method was applied to correct the alpha level and *P* values accordingly.

## Results

### Demographic and Clinical Characteristics of Participants

Characteristics of 1122 participants were shown in Table 1. No significant statistical difference was observed for age and gender between PD and controls (*P* > 0.05). Compared to controls, PD patients were less likely to ever smoke cigarettes or drink alcohol (*P* < 0.05) (Table 1), which is consistent with several studies (Noyce et al., 2012; Zhang et al., 2014). Gender, disease duration, Hoehn and Yahr stage and oral LED/day showed different distribution among three PD motor subtypes: There were more male patients in the AR group; Tremor Dominant (TD) group consisted of more early stage PD patients compared to AR and MX group. Between EOPD and LOPD subgroups, the distribution of age and age at onset was as expected (Table 1).

**Table 1:** Demographic and clinical characteristics of controls and PD patients.

Characteristics^a^	Controls *N* = 599	Total PD *N* = 523	*p*	Subtypes of PD defined by motor symptoms				Subtypes of PD defined by onset age		
				TD *n* = 152	AR *n* = 225	MX *n* = 146	*p*	EOPD *n* = 99	LOPD *n* = 424	*p*
Gender										
Male	322 (53.8)	288 (55.1)	0.660	73 (48.0)	137 (60.9)	78 (53.4)	**0.043**	60 (60.6)	228 (53.8)	0.218
Female	277 (46.2)	235 (44.9)		79 (52.0)	88 (39.1)	68 (46.6)		39 (39.4)	196 (46.2)	
Age	63.94 ± 10.05	63.13 ± 9.51	0.388	63.48 ± 9.54	62.94 ± 9.70	63.07 ± 9.21	0.893	50.61 ± 8.24	66.06 ± 7.11	**<0.001**
Age at onset	NA	58.42 ± 9.76	NA	59.20 ± 9.77	58.45 ± 9.93	57.78 ± 9.48	0.670	44.06 ± 5.82	61.85 ± 7.05	**<0.001**
Disease duration (year)	NA	4.52 ± 4.29	NA	3.51 ± 3.87	4.66 ± 4.63	5.36 ± 3.96	**<0.001**	6.32 ± 6.20	4.10 ± 3.59	**0.003**
Hoehn and Yahr stage										
1–1.5	NA	233 (46.8)	NA	78 (52.0)	101 (48.6)	54 (38.6)	**0.025**	38 (40.8)	195 (48.1)	0.248
2–2.5	NA	193 (38.7)		60 (40.0)	75 (36.1)	58 (41.4)		37 (39.8)	156 (38.6)	
≥ 3	NA	72 (14.5)		12 (8.0)	32 (15.4)	28 (20.0)		18 (19.4)	54 (13.3)	
Oral LED/day (mg)	NA	366.64 ± 287.7	NA	263.61 ± 234.14	397.34 ± 299.85	423.87 ± 292.24	**<0.001**	424.46 ± 333.15	352.58 ± 274.23	0.104
Smokers (Yes/No)	163/436	74/449	**<0.001**	20/132	42/183	12/134	**0.017**	19/80	55/369	0.110
Alcohol drinkers (Yes/No)	102/497	62/461	**0.014**	14/138	35/190	13/133	0.075	12/87	50/374	0.927

*TD, tremor dominant subtype of Parkinson’s disease; AR, Akinetic/Rigid dominant subtype of Parkinson’s disease; MX, mixed subtype of Parkinson’s disease; EOPD, early onset Parkinson’s disease; LOPD, late onset Parkinson’s disease; SD, standard deviation; NA, not available. *P*-values that reach statistical significance were shown in bold. ^*a*^Attributes data are presented as mean ± SD; variables data are presented as numbers (%).*

### Lack of Association Between DJ-1 Promoter Polymorphisms and PD in Single-Point Analysis

Three single nucleotide polymorphisms (SNPs) and one 18 bp deletion were observed in our cohort. Each of them already has a reference in the SNP database of NCBI (rs17523802, rs226249, rs200968609, and rs35675666). Schematic view of relative positions of these polymorphisms according to TSS was shown in Figure 1. All the four variations were in HWE among PD and controls (*P* > 0.001). There was no statistical difference in genotype or allele distribution of the four variations between the entire PD group and control (Table 2). When patients were stratified by life style factors (cigarette or alcohol intake), no significance of allele or genotype distribution was observed in all subgroups for the four variations (data not shown). Stratification analysis of age or gender also showed no statistical differences between PD and control among the four variations after Bonferroni adjustment (data not shown).

**Table 2:** Genotype and allele distribution between total PD patients and controls of polymorphisms in DJ-1 promoter region.

SNP ID^a^	Position^b^	Allele/Genotype	PD (*n* = 523)	Control (*n* = 599)	*p^c^*	OR (95% CI)^c^
Rs17523802	–21	G	982 (93.9)	1107 (92.4)	0.221	0.806 (0.570,1.139)
		A	64 (6.1)	91 (7.6)		
		GG	460 (87.9)	510 (85.2)	0.215	0.800 (0.562,1.139)
		GA	62 (11.9)	87 (14.5)		
		AA	1 (0.2)	2 (0.3)		
Rs226249	18	C	681 (65.1)	802 (66.9)	0.123	1.155 (0.962,1.386)
		T	365 (34.9)	396 (33.1)		
		CC	232 (44.4)	274 (45.7)	0.131	1.147 (0.96,1.369)
		CT	217 (41.5)	254 (42.4)		
		TT	74 (14.1)	71 (11.9)		
Rs200968609	168_185del	Ins	982 (93.9)	1107 (92.4)	0.221	0.806 (0.570,1.139)
		Del	64 (6.1)	91 (7.6)		
		Ins/ins	460 (87.9)	510 (85.2)	0.215	0.800 (0.562,1.139)
		Ins/del	62 (11.9)	87 (14.5)		
		Del/del	1 (0.2)	2 (0.3)		
Rs35675666	213	G	982 (93.9)	1107 (92.4)	0.221	0.806 (0.570,1.139)
		T	64 (6.1)	91 (7.6)		
		GG	460 (87.9)	510 (85.2)	0.215	0.800 (0.562,1.139)
		GT	62 (11.9)	87 (14.5)		
		TT	1 (0.2)	2 (0.3)		

*OR, odds ratio; 95% CI, 95% confidence interval. ^*a*^SNP ID represents the ID of each polymorphism recorded in the NCBI SNP database. ^*b*^The first base of TSS was defined as 1, the first upstream base of TSS is -1, the relative position of each polymorphism was calculated based on TSS. ^*c*^Adjusted for age, gender, cigarettes, and alcohol.*

To investigate the association between polymorphisms and PD clinical phenotypes, genotype and allele distribution analysis between control and clinical subtypes of PD were further conducted. However, no significant difference was reached on either the genotype or allele distribution of the four variations among the entire control group and the motor subtypes of PD or among the EOPD (age of onset < 50), LOPD (age of onset ≥ 50) group and control group (Table 3).

**Table 3:** Allele distribution of DJ-1 promoter polymorphisms between controls and different PD subtypes classified by motor features or age at onset.

SNP ID	Allele/ genotype		Motor subtype of PD			AR vs. TD		Onset-age PD subtypes		EOPD vs. LOPD	
		Control	TD	AR	MX	*p*^a^	OR (95% CI)^a^	EOPD	LOPD	*p*^a^	OR (95% CI)^a^
		*n* = 599	*n* = 152	*n* = 225	*n* = 146			*n* = 99	*n* = 424		
rs17523802	G	1107 (92.4)	277 (91.1)	420 (93.3)	277 (94.9)	0.312	0.75 (0.44,1.3)	186 (93.9)	794 (93.9)	0.221	0.81 (0.57,1.14)
	A	91 (7.6)	27 (8.9)	30 (6.7)	15 (5.1)			12 (6.1)	52 (6.1)		
	GG	510 (85.2)	125 (82.2)	196 (87.1)	131 (89.7)	0.299	0.74 (0.42,1.3)	87 (87.9)	373 (88.0)	0.457	0.67 (0.24,1.92)
	GA	87 (14.5)	27 (17.8)	28 (12.4)	15 (10.3)			12 (12.1)	50 (11.8)		
	AA	2 (0.3)	0 (0)	1 (0.4)	0 (0)			0 (0)	1 (0.2)		
rs226249	C	802 (66.9)	200 (65.8)	285 (63.3)	196 (67.1)	0.525	1.11 (0.81,1.51)	136 (68.7)	544 (64.3)	0.12	1.16 (0.96,1.39)
	T	396 (33.1)	104 (34.2)	165 (36.7)	96 (32.9)			62 (31.3)	302 (35.7)		
	CC	274 (45.7)	64 (42.1)	99 (44)	69 (47.3)	0.540	1.10 (0.82,1.48)	50 (50.5)	182 (42.9)	0.88	1.04 (0.65,1.66)
	CT	254 (42.4)	72 (47.4)	87 (38.7)	58 (39.7)			36 (36.4)	181 (42.7)		
	TT	71 (11.9)	16 (10.5)	39 (17.3)	19 (13)			13 (13.1)	61 (14.4)		
rs200968609	Ins	1107 (92.4)	277 (91.1)	420 (93.3)	277 (94.9)	0.312	0.75 (0.44,1.3)	186 (93.9)	794 (93.9)	0.221	0.81 (0.57,1.14)
	Del	91 (7.6)	27 (8.9)	30 (6.7)	15 (5.1)			12 (6.1)	52 (6.1)		
	Ins/ins	510 (85.2)	125 (82.2)	196 (87.1)	131 (89.7)	0.299	0.74 (0.42,1.3)	87 (87.9)	373 (88.0)	0.45	0.67 (0.24,1.92)
	Ins/del	87 (14.5)	27 (17.8)	28 (12.4)	15 (10.3)			12 (12.1)	50 (11.8)		
	Del/del	2 (0.3)	0 (0)	1 (0.4)	0 (0)			0 (0)	1 (0.2)		
rs35675666	G	1107 (92.4)	277 (91.1)	420 (93.3)	277 (94.9)	0.312	0.75 (0.44,1.3)	186 (93.9)	794 (93.9)	0.22	0.81 (0.57,1.13)
	T	91 (7.6)	27 (8.9)	30 (6.7)	15 (5.1)			12 (6.1)	52 (6.1)		
	GG	510 (85.2)	125 (82.2)	196 (87.1)	131 (89.7)	0.299	0.74 (0.42,1.3)	87 (87.9)	373 (88.0)	0.46	0.67 (0.24,1.92)
	GT	87 (14.5)	27 (17.8)	28 (12.4)	15 (10.3)			12 (12.1)	50 (11.8)		
	TT	2 (0.3)	0 (0)	1 (0.4)	0 (0)			0 (0)	1 (0.2)		

*TD, tremor dominant subtype of Parkinson’s disease; AR, Akinetic/Rigid dominant subtype of Parkinson’s disease; MX, mixed subtype of Parkinson’s disease; EOPD, early onset Parkinson’s disease; LOPD, late onset Parkinson’s disease. ^*a*^Adjusted for age, gender, cigarettes and alcohol.*

### Lack of Association Between DJ-1 Promoter Polymorphisms and PD in Haplotype Analysis

Since the four detected polymorphisms were located on the same chromosome, to explore whether they were in linkage disequilibrium (LD) linkage analysis was performed. Strong linkage patterns were observed among rs17523802, rs200968609, and rs35675666) (*r*^2^ = 1.0, D’ = 1.0, LOD = 206.52). The four variations constitute one block of haplotype. Three Haplotypes with a frequency greater than 1% in all samples (G-T-ins-G, G-C-ins-G, and A-C-del-T, alleles arrayed in order of rs17523802, rs226249, rs200968609, and rs35675666) were selected to analyze. However, the frequency of the three Haplotypes showed no difference between PD and control (Table 4).

**Table 4:** Haplotype frequencies of the four variations in DJ-1 promoter region.

	Haplotype^a^	Total	PD	control	*p*	OR (95% CI)
		2N = 2244	2n = 1046	2n = 1198		
Block 1	1. G-C-ins-G	1320 (58.8)	609 (58.2)	711 (59.3)	0.588	0.96 (0.81, 1.13)
	2. G-T-ins-G	761 (33.9)	365 (34.9)	396 (33.1)	0.358	1.09 (0.91, 1.29)
	3. A-C-del-T	163 (7.3)	72 (6.9)	91 (7.6)	0.516	0.90 (0.65, 1.24)

*OR, odds ratio; 95% CI, 95% confidence interval. ^*a*^Alleles in haplotype are arrayed in order of rs17523802, rs226249, rs200968609, and rs35675666.*

### Analyzing Effects of the Four Variations on PD With Public GWAS Database

To further evaluate the effect of these polymorphisms on PD, we looked up these polymorphisms in existing public genomic databases. As shown in Table 5, MAF of the four variations in our study were quite in accordance with the MAFs of East Asian population in 1000 Genome Project database, and lower than the MAFs of all population in either 1000 Genome Project database or TOPMED program database. We searched the meta-analysis results based on 13,708 PD cases and 95,282 controls from 13 independent GWAS datasets of European descent in PDGene database. Due to data sharing restrictions, detailed genotype information of the four variations were not accessible. Thus, only Meta *p* values and Meta OR of the four variations in GWAS reports were listed in Table 5. All the four variations showed Meta *P*-value > 0.05, which suggested, not only in Chinese as this study observed, the four variations may also not be associated with PD in European populations.

**Table 5:** The frequencies of polymorphisms detected in present study and in public databases.

Polymorphisms	Minor allele frequency (%)							
	Present study		dbsnp147 database	1000 Genomes Project database		TOPMED program database	PDGene database	
	PD	Control		(All population)	(East Asian)		Meta *P*-value^a^	Meta OR^a^
–21 G > A	6.1	7.6	rs17523802	17.65	7	22.33	>0.05	>1
18 C > T	34.9	33.1	rs226249	36.26	33	29.18	>0.05^§^	>1^§^
168_185del	6.1	7.6	rs200968609	9.29	7	–	>0.05^§^	>1^§^
213 G > T	6.1	7.6	rs35675666	14.82	7	18.36	>0.05	>1

*TOPMED, Trans-Omics for Precision Medicine Program. ^*a*^The meta-analysis includes data up to 13,708 PD cases and 95,282 control from 15 independent GWAS datasets. The meta *p*-value ≥ 0.05 corresponds to *p*-values ≤ 1 and ≥0.05. ^§^ rs226249 and rs200968609 had no record in the database; Since rs226249 was in LD with rs2493215 (*r*^*2*^ = 0.896 in 1000 Genome), we looked up rs2493215 to represent rs226249 in PDGene and found a Meta *p*-value > 0.05 with Meta OR > 1; considering rs200968609 was in LD with rs17523802 and rs35675666 (*r*^*2*^ = 0.896 in 1000 Genome), the results of rs17523802 and rs35675666 could also represent that of rs200968609.*

### Meta-Analysis of DJ-1 Promoter Polymorphisms Based on This Study and Other Previous Reports

**FIGURE 2:**
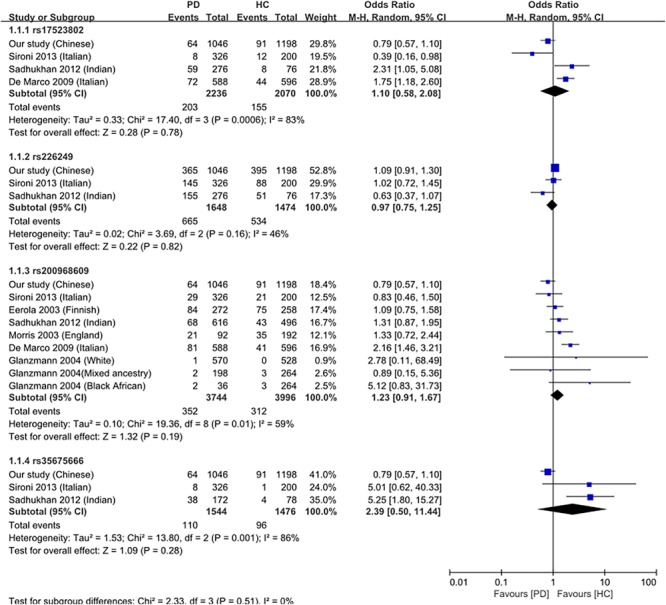
Forest plots of the meta-analysis between DJ-1 promoter polymorphisms and PD under the allelic model. For meta-analysis, rs17523902 *p* = 0.777, rs226249 *p* = 0.816, rs200968609 *p* = 0.188, and rs35675666 *p* = 0.276. OR, odds ratio; CI, confidence interval; I-squared, heterogeneity.

Except for meta-analysis on GWAS reports, a meta-analysis of the four variations with PD was performed based on this study and other related previous case-control studies (Eerola et al., 2003; Morris et al., 2003; De Marco et al., 2010; Sadhukhan et al., 2012; Sironi et al., 2013; Glanzmann et al., 2014). Detailed information of previous studies was shown in Table 6. Consistent with our results, no significant associations were observed between these polymorphisms and PD (rs17523902 *p* = 0.777, rs226249 *p* = 0.816, rs200968609 *p* = 0.188, and rs35675666 *p* = 0.276) at the allelic level under the random effect model (Figure 2), which indicated that DJ-1 promoter polymorphisms may play little role in PD susceptibility in different ethnic populations.

**Table 6:** Detailed information of previous studies selected into meta-analysis.

				PD		Control				
Polymorphisms	Chr	Pos (hg38)	SNP ID	MAF (%)	Cases	MAF (%)	Cases	*P*-value	Ethnic background	Study
–21 G > A	1	7961680	rs17523802	2.5	163EOPD	6	100	0.039^a^	Italian	Sironi et al., 2013
				21.4	138PD	10.5	38	0.033^§^	Indian	Sadhukhan et al., 2012
				12.2	294PD	7.3	298	0.005	Italian	De Marco et al., 2010
18 C > T	1	7961718	rs226249	44.5	163EOPD	44	100	0.915^a^	Italian	Sironi et al., 2013
				56.2	138PD	67.1	38	0.086^§^	Indian	Sadhukhan et al., 2012
168_185del	1	7961913	rs200968609	8.9	163EOPD	10.5	100	0.543^a^	Italian	Sironi et al., 2013
				31	136sporadic PD	29	129	0.65	Finnish	Eerola et al., 2003
				11	308PD	8.7	248	0.19^§^	Indian	Sadhukhan et al., 2012
				23	46PD	18	96	0.362^§^	England	Morris et al., 2003
				13.8	294PD	6.9	298	<0.001	Italian	De Marco et al., 2010
				0.2	285PD	0	264	0.497	White	Glanzmann et al., 2014
				1	99PD	1.1	132	0.337	Mixed ancestry	Glanzmann et al., 2014
				5.6	18PD	1.1	132	0.111	Black African	Glanzmann et al., 2014
213 G > T	1	7961850	rs35675666	2.5	163EOPD	0.5	100	0.193^a^	Italian	Sironi et al., 2013
				22.1	86PD	5.1	39	<0.001^§^	Indian	Sadhukhan et al., 2012

*Chr, chromosome; pos (hg38), SNP position on hg38 reference sequence; MAF, minor allele frequency. ^*a*^Values were non-significant according to Bonferroni correction (*p* < 0.002). ^§^*P* values calculated with provided data in the studies (which were not directly provided in the studies).*

### Effects of Promoter Polymorphisms on DJ-1 Promoter Transcriptional Activity

**FIGURE 3:**
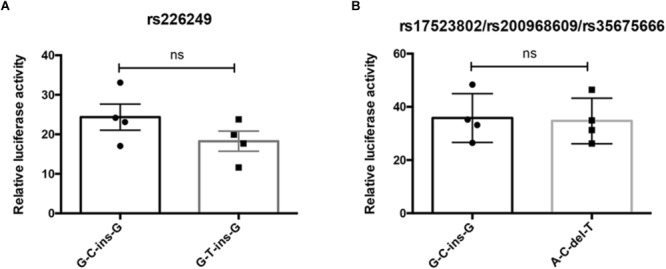
The influence of promoter polymorphisms on DJ-1 promoter transcriptional activity. Dual-luciferase reporter assay was used to access whether rs226249 **(A)** or rs17523802/rs200968609/rs35675666 **(B)** affect DJ-1 promoter transcriptional activity. The haplotype alleles arrayed in order of rs17523802, rs226249, rs200968609, and rs35675666. The data were represented as mean ± SE from four independent transfection experiments, each in duplicate.

To test whether the four variations alter DJ-1 promoter transcriptional activity, dual-luciferase reporter gene assay was conducted. As shown in Figure 3, allele alteration of rs226249 or rs17523802/rs200968609/rs35675666 had no effect on DJ-1 promoter transcriptional activity.

### EQTL (Expression Quantitative Trait Loci) Analysis of the Four Variations

**FIGURE 4:**
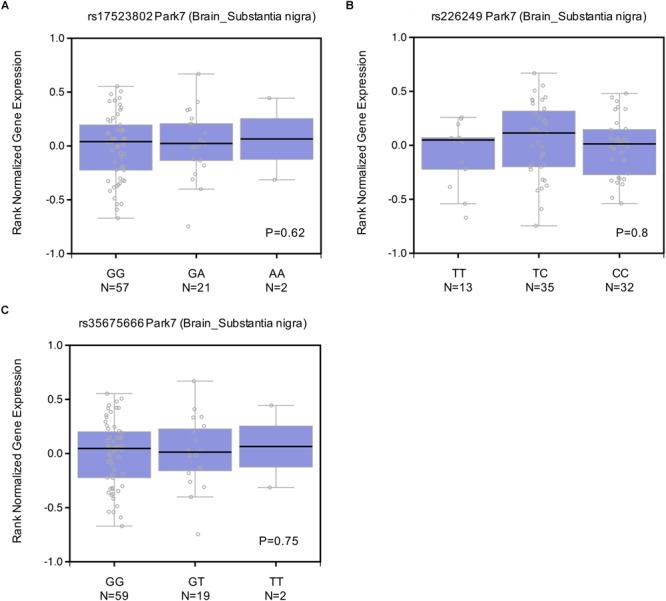
Effect of rs17523802 **(A)**, rs226249 **(B)**, rs356756666 **(C)** genotype on DJ-1 mRNA expression in normal human brain substantia nigra. Data of rs200968609 were not found in dbGap.

To explore whether these DJ-1 promoter polymorphisms could impact human brain DJ-1 gene expression, eQTL analysis was conducted with data from dbGaP. As shown in Figure 4, rs17523802 (*P* = 0.62), rs226249 (*P* = 0.8), and rs35675666 (*P* = 0.75) showed no association with DJ-1 gene expression in human brain substantia nigra. In addition, other brain regions including amygdala, anterior cingulate cortex (BA24), Caudate (basal ganglia), Frontal Cortex (BA9), Hippocampus, Hypothalamus, nucleus accumbens (basal ganglia), putamen (basal ganglia), spinal cord (cervical c-1) were also analyzed and found negative results (Table 7).

**Table 7:** Effect of rs17523802, rs226249, and rs356756666 on DJ-1 mRNA expression in different regions of normal human brain.

Polymorphisms	Gene	dbSNP number	Tissue	*P*-value	Effect size
–21 G > A	PARK7	rs17523802	Brain–Amygdala	0.19	–0.1
			Brain–Anterior cingulate cortex (BA24)	0.23	–0.089
			Brain–Caudate (basal ganglia)	0.81	–0.016
			Brain–Frontal Cortex (BA9)	0.18	–0.084
			Brain–Hippocampus	0.097	–0.092
			Brain–Hypothalamus	0.065	–0.11
			Brain–Nucleus accumbens (basal ganglia)	0.4	–0.052
			Brain–Putamen (basal ganglia)	0.55	0.047
			Brain–Spinal cord (cervical c-1)	0.38	0.066
			Brain–Substantia nigra	0.62	0.045
18 C > T	PARK7	rs226249	Brain–Amygdala	0.48	0.043
			Brain–Anterior cingulate cortex (BA24)	0.84	0.013
			Brain–Caudate (basal ganglia)	0.26	–0.06
			Brain–Frontal Cortex (BA9)	0.22	0.071
			Brain–Hippocampus	0.35	–0.044
			Brain–Hypothalamus	0.38	0.049
			Brain–Nucleus accumbens (basal ganglia)	0.51	0.032
			Brain–Putamen (basal ganglia)	0.33	–0.058
			Brain–Spinal cord (cervical c-1)	0.68	0.027
			Brain–Substantia nigra	0.8	–0.017
213 G > T	PARK7	rs35675666	Brain–Amygdala	0.27	–0.089
			Brain–Anterior cingulate cortex (BA24)	0.24	–0.087
			Brain–Caudate (basal ganglia)	0.64	–0.032
			Brain–Frontal Cortex (BA9)	0.11	–0.099
			Brain–Hippocampus	0.2	–0.072
			Brain–Hypothalamus	0.073	–0.11
			Brain–Nucleus accumbens (basal ganglia)	0.46	–0.046
			Brain–Putamen (basal ganglia)	0.61	0.041
			Brain–Spinal cord (cervical c-1)	0.4	0.064
			Brain–Substantia nigra	0.75	0.029

## Discussion

The antioxidative effect of DJ-1 plays an important role in cell survival, deficiency or low level of DJ-1 protein makes neurons more susceptible to oxidative stress and result in the occurrence of PD. In familial PD, such deficiency is caused by mutations in DJ-1 gene, whereas in sporadic PD, who lacks mutations in DJ-1, the reason of a low brain DJ-1 protein level remains obscure. This study attempted to reveal whether polymorphisms in DJ-1 promoter were associated with PD through affecting the regulation of DJ-1 expression. In the present study, we sequenced the core region of DJ-1 promoter in 523 sporadic PD patients and 599 controls in Chinese Han population of mainland, and finally found four variations (rs17523802, rs226249, rs200968609, and rs35675666). However, no significant association was found between all the four detected polymorphisms and the risk of PD either in the allelic model or genotype model, in single-point analysis or haplotype analysis. After stratification by age, gender, PD subtypes or habitude of cigarette or alcohol, we still failed to find any difference in the distribution of the four variations between PD and control or among subtypes. Searching results in PDGene GWAS database and meta-analysis of the present study and previous reports also showed no association between the four variations and PD. In addition, eQTL analysis demonstrated lack of association between the four DJ-1 promoter polymorphisms and normal human brain DJ-1 gene expression. All of these results suggested DJ-1 promoter polymorphisms may play little role in regulating brain DJ-1 gene expression level and PD susceptibility.

To our best knowledge, this is the first survey of the association between DJ-1 promoter polymorphisms and PD risk in a relatively large sample size of Chinese sporadic PD patients. Previous reports on DJ-1 promoter polymorphisms are very rare, and the results are not consistent. De Marco et al. (2010) found rs17523802 (*P* = 0.005) and rs200968609 (*P* < 0.001) were associated with PD risk in an Italian cohort (294PD and 298 control) (Table 6). Whereas, Sadhukhan et al. (2012) suggested rs35675666 (*P* < 0.001) was a risk factor of PD in an Indian population (86PD and 39 control), and rs17523802 and rs200968609 were not associated with PD (Table 6). Other studies (Eerola et al., 2003; Morris et al., 2003; Sironi et al., 2013; Glanzmann et al., 2014) failed to find association between these polymorphisms and PD (Table 6). Meta-analysis of previous reports and our present study suggested no association between the four variations and PD. The results of GWAS database and eQTL analysis further confirmed that polymorphisms in DJ-1 promoter region (rs17523802, rs226249, rs200968609, and rs35675666) were not associated with DJ-1 expression in human brain and the risk of PD.

Based on current findings, low DJ-1 protein level in sporadic PD patients is not a consequence of DJ-1 promoter polymorphisms. According to relevant researches, the low DJ-1 protein level in sporadic PD might be related to dysregulation of gene expression. On the transcriptional level, it might be caused by altered regulation of transcriptional factors or epigenetic regulation such as DNA methylation and histone modifications. Taira et al. (2001) and Duplan et al. (2013), respectively, found the transcription factor SP-1 (Taira et al., 2001) or XBP-1S (Duplan et al., 2013) could bind to the DJ-1 promoter region and increase its transcriptional activity, suggesting that expression or activity alterations of transcriptional factors might be associated with PD. Zhou et al. (2011) demonstrated that deacetylase inhibitors such as phenyl butyrate and sodium butyrate could increase DJ-1 mRNA and protein expression to protect cells against oxidative stress, which suggested alterations of acetylation of histone for DJ-1, might affect DJ-1 expression. However, in our previous study (Tan et al., 2016), we found that DNA methylation did not regulate DJ-1 expression. On the post-transcriptional level, microRNAs may have an effect on the expression of DJ-1 protein level. Our previous study (Chen et al., 2017) found that MircoRNA-4639 could downregulate DJ-1 expression and had the potential to be a biomarker for PD. Thus, low DJ-1 protein levels in sporadic PD might be a combination result of a variety of factors, future studies on detailed mechanisms would be needed.

In conclusion, our results implicated that DJ-1 promoter polymorphisms may not be associated with PD risk.

## Author Contributions

JD designed the project. SC, YW, and YT collected the samples. LH, SL, RS, HP, MW, ZL, and SS conducted genotyping and analyzed the data. JD, LH, and SC wrote the manuscript. All authors have approved the final version of the manuscript.

## Conflict of Interest Statement

The authors declare that the research was conducted in the absence of any commercial or financial relationships that could be construed as a potential conflict of interest.

## Footnotes

1 snapgene.com

2 softgenetics.com

3 http://www.pdgene.org

4 https://www.gtexportal.org

5 broadinstitute.org
